# Differences among COVID-19, Bronchopneumonia and Atypical Pneumonia in Chest High Resolution Computed Tomography Assessed by Artificial Intelligence Technology

**DOI:** 10.3390/jpm11050391

**Published:** 2021-05-10

**Authors:** Robert Chrzan, Monika Bociąga-Jasik, Amira Bryll, Anna Grochowska, Tadeusz Popiela

**Affiliations:** 1 Department of Radiology, Jagiellonian University Medical College, Kopernika 19, 31-501 Krakow, Poland; bryllamira@gmail.com (A.B.); agrochowska@su.krakow.pl (A.G.); tadeusz.popiela@uj.edu.pl (T.P.); 2 Department of Infectious Diseases, Jagiellonian University Medical College, Jakubowskiego 2, 30-688 Krakow, Poland; monika.bociaga-jasik@uj.edu.pl

**Keywords:** COVID-19, HRCT, artificial intelligence, ground glass

## Abstract

The aim of this study was to compare the results of automatic assessment of high resolution computed tomography (HRCT) by artificial intelligence (AI) in 150 patients from three subgroups: pneumonia in the course of COVID-19, bronchopneumonia and atypical pneumonia. The volume percentage of inflammation and the volume percentage of “ground glass” were significantly higher in the atypical (respectively, 11.04%, 8.61%) and the COVID-19 (12.41%, 10.41%) subgroups compared to the bronchopneumonia (5.12%, 3.42%) subgroup. The volume percentage of consolidation was significantly higher in the COVID-19 (2.95%) subgroup compared to the atypical (1.26%) subgroup. The percentage of “ground glass” in the volume of inflammation was significantly higher in the atypical (89.85%) subgroup compared to the COVID-19 (79.06%) subgroup, which in turn was significantly higher compared to the bronchopneumonia (68.26%) subgroup. HRCT chest images, analyzed automatically by artificial intelligence software, taking into account the structure including “ground glass” and consolidation, significantly differ in three subgroups: COVID-19 pneumonia, bronchopneumonia and atypical pneumonia. However, the partial overlap, particularly between COVID-19 pneumonia and atypical pneumonia, may limit the usefulness of automatic analysis in differentiating the etiology. In our future research, we plan to use artificial intelligence for objective assessment of the dynamics of pulmonary lesions during COVID-19 pneumonia.

## 1. Introduction

At the beginning of the COVID-19 pandemic, high hopes were placed for using diagnostic imaging, in particular chest HRCT (high resolution computed tomography), to detect positive cases. However, it turned out that the specificity is limited, because the symptoms observed may occur in infections of other etiologies.

According to the current recommendations of radiological societies (British Thoracic Imaging Society [1], American College of Radiology [2]), chest HRCT should not be used as a screening tool or as a first-line test in the diagnosis of COVID-19 infection. A PCR (polymerase chain reaction) test of a nasopharyngeal or pharyngeal swab remains the basic method of verifying COVID-19. However, chest HRCT is now often used to assess the complications in COVID-19 patients.

Artificial intelligence technology is currently of great interest in medical imaging. Software that automatically recognizes abnormal structures on X-rays or CT images can be a very valuable aid in the daily work of a radiologist [3,4].

In the course of the pandemic, the main reason for the interest in the assessment of HRCT images by artificial intelligence was the ability to analyze large numbers of patients in a short time [5], which was particularly important in centers with an insufficient number of qualified radiologists. However, the question quickly arose as to whether such technology could accurately identify COVID-19 pneumonia and differentiate it from pneumonia of a different etiology [5,6].

Symptomatic COVID-19 patients typically have respiratory and systemic manifestations. COVID-19 pneumonia most commonly presents with fever, cough, sputum production and shortness of breath. The primary findings in HRCT are: “ground glass” opacities (usually bilateral, peripheral and subpleural), crazy paving pattern, consolidations and bronchovascular thickening [7,8].

Bronchopneumonia, also known as lobular pneumonia, is the most common type of pneumonia, caused by bacteria, such as *Staphylococcus aureus*, *Klebsiella pneumoniae*, *Haemophilus influenzae, Pseudomonas aeruginosa, and Escherichia coli*, and less often anaerobes, such as *Proteus* species. Clinically, bronchopneumonia may present with productive cough, malaise, dyspnea, fever, shivers, pleuritic pain and occasionally hemoptysis. Suppurative peribronchiolar inflammation and subsequent patchy consolidation of one or more secondary lung lobules create an HRCT pattern of multiple focal opacities in a lobular pattern, centered at centrilobular bronchioles (a tree-in-bud appearance) [9]. Such small foci can merge into larger heterogeneous confluent areas of consolidation.

“Atypical” pneumonia is a type of pneumonia that is not caused by one of the pathogens most commonly associated with the disease and with clinical presentation different from “typical” pneumonia. The most common microorganisms that cause atypical pneumonia are: “atypical” bacteria, such as *Mycoplasma pneumoniae*, *Chlamydia pneumoniae*, *Chlamydia psittaci, Coxiella burnetii, Francisella tularensis, and Legionella pneumophila*, and viruses, such as respiratory syncytial virus (RSV), influenza A and B, parainfluenza, adenovirus, severe acute respiratory syndrome (SARS), Middle East respiratory syndrome (MERS), measles or even fungi and protozoa. It is clinically characterized by a low-grade fever, a moderate amount of sputum, a persistent dry cough, more pronounced general symptoms, such as a headache and myalgia, no response to sulfonamide and beta-lactams, no leukocytosis, no symptoms of lobar consolidation and lack of alveolar exudate. The inflammation is usually limited to the pulmonary interstitium and the interlobular septa; therefore, atypical pneumonia usually has a pattern of focal or diffuse bilateral “ground glass” opacities in HRCT [9].

The aim of the study was to compare the results of automatic assessment of HRCT images by a software, using artificial intelligence technology, in three subgroups of patients: with pneumonia in the course of COVID-19, with bronchopneumonia and with “atypical” pneumonia.

A new idea in our research was to use artificial intelligence, not to simply distinguish COVID-19 pneumonia from pneumonia of other causes, but to automatically detect pulmonary lesions and assess their structure, including the areas of “ground glass” and consolidations, to finally compare such structure in three subgroups of pneumonia as above.

## 2. Materials and Methods

The HIS (hospital information system) and the hospital PACS (picture archiving and communication system) were retrospectively searched for patients with clinical symptoms of pneumonia confirmed in chest HRCT examinations.

Patients with pre-existing chronic lung diseases, including severe emphysema and interstitial lung diseases, were excluded from the study.

Three subgroups of patients were analyzed:

1. The patients with pneumonia in the course of COVID-19 infection were confirmed by PCR from nasopharyngeal swabs. Fifty patients from the period of 06.04.2020–12.06.2020 were selected, which resulted in a subgroup consisting of 18 females, 32 males, 34–86 years old, average of 60 years old;
2. The patients with bronchopneumonia were diagnosed both in the patients’ clinical documentation and radiological assessment. Fifty patients from the period of 11.03.2013–03.06.2020 were selected, which resulted in a subgroup consisting of 20 females, 30 males, 28–97 years old, average of 65 years old;
3. The patients with “atypical” pneumonia were diagnosed both in the patients’ clinical documentation and radiological assessment. Fifty patients from the period of 03.09.2010–09.01.2020 were selected, which resulted in a subgroup consisting of 18 females, 32 males, 20–80 years old, average of 55 years old. According to clinical data, the virus was the etiological factor in 21 cases, including 4 cases of influenza, 2 cases of cytomegalovirus (CMV), 1 case of respiratory syncytial virus (RSR) and the remaining 14 cases were not specified; *Pneumocystis jiroveci* (PCP) in 8 cases; *Mycoplasma pneumoniae* in 1 case; *Chlamydia trachomatis* in 1 case; and in the remaining 19 cases of clinically and radiologically diagnosed atypical pneumonia, no etiological factor was specified in the available documentation.

All the HRCT examinations were performed using multirow (64 or 80 rows) helical scanners, slice thickness of 0.625 mm, 0.75 mm, 1 mm or 1.25 mm, mainly 120 kV, 100–350 mAs.

**Figure 1:**
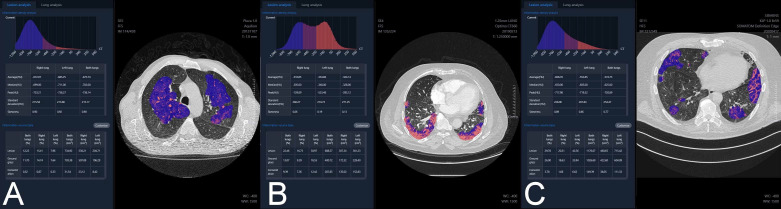
The examples of analysis of (**A**) atypical pneumonia, (**B**) bronchopneumonia and (**C**) COVID-19 pneumonia.

For every patient, the HRCT images in original DICOM (Digital Imaging and Communications in Medicine) format were transferred from PACS to the processing server YITU Healthcare set up and configured in the local hospital network. The server software was developed by YITU CT, YITU Healthcare Technology Co., Ltd. (Shanghai, China) in cooperation with Huawei Technologies Co., Ltd., China (Shenzhen, China) [10,11]. The images were automatically analyzed by the software, using artificial intelligence technology. The examples of analysis of atypical pneumonia, bronchopneumonia and COVID-19 pneumonia are presented in Figure 1.

**Figure 2:**
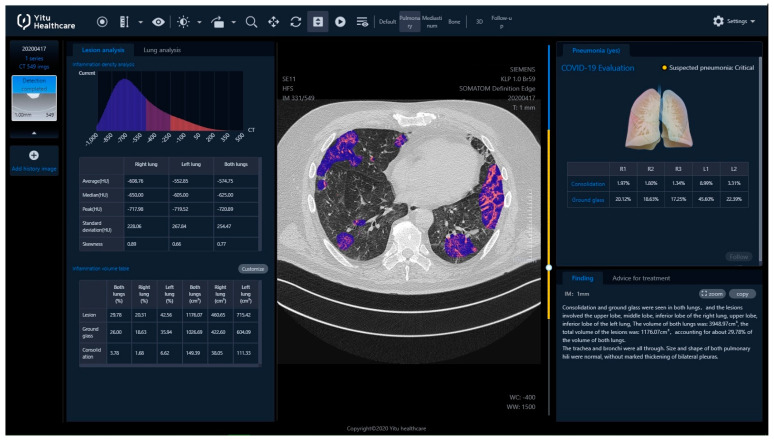
The final report from automatic assessment of HRCT images by the software, using artificial intelligence technology.

The final report from the assessment was available after login into the server (Figure 2).

The report included, i.a.:

- Inflammation CT density histogram, the values of average, median, and peak CT density for the inflammation regions in the right, the left and both lungs;
- Inflammation (“**Lesion**”) volume, in cubic centimeters and as a percentage of the whole lung tissue, for the right, the left and both lungs;
- Within the inflammation volume as above, distinction into “**Ground glass**” and “**Consolidation**” (**Lesion** = **Ground glass** + **Consolidation**), in cubic centimeters and as a percentage of the whole lung tissue, for the right, the left and both lungs;
- The HRCT images with regions of inflammation marked in color;
- Inflammation volume as a percentage of the whole lung tissue, with distinction into “**Ground glass**” and “**Consolidation**”, independently for every lung lobe, additionally with a “pseudo 3D” graphic presentation;
- Estimated risk of pneumonia, assessed by artificial intelligence software based on CT evaluation, reported as mild, moderate or critical (“**Suspected pneumonia**”);
- Preprepared text of radiological report.

It was also possible to export all the assessment results from the processing server to the Excel spreadsheet.

For the purposes of this study, the following parameters were analyzed in every patient: **Lesion volume Both lungs (%)**, the volume of inflammation (“**Lesion**”) as a percentage of the volume of both lungs; **Ground glass volume Both lungs (%)**, the volume of “**Ground glass**” as a percentage of the volume of both lungs; **Consolidation volume Both lungs (%)**, the volume of consolidation as a percentage of the volume of both lungs; **Ground glass volume/Lesion volume Both lungs (%)**, the volume of “**Ground glass**” as a percentage of the volume of inflammation (“**Lesion**”) in both lungs; and **Suspected pneumonia**, the estimated risk of pneumonia.

Statistical software R 4.0.3 (Vienna, Austria) [12] was used for computations by a certified statistician.

The comparison of the values of quantitative variables in three groups was performed using the Kruskal–Wallis test. After detecting statistically significant differences, post-hoc analysis with Dunn’s test was performed to identify significantly different groups.

The comparison of the values of qualitative variables in the groups was performed using the chi-squared test.

The significance level for all statistical tests was set to 0.05.

The study was approved by the local bioethics committee (opinion No. 1072.6120.333.2020 of 7 December 2020).

## 3. Results

**Figure 3:**
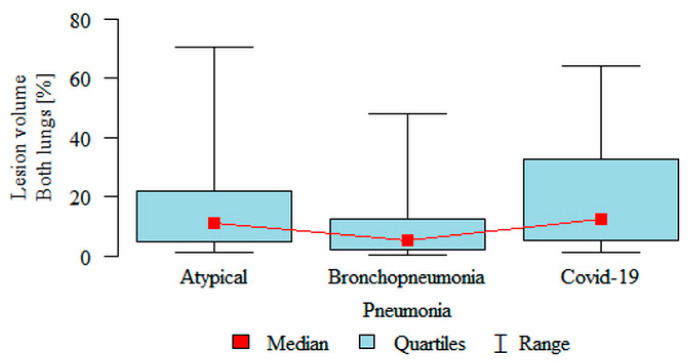
Comparison of the volume of inflammation (“lesion”) as a percentage of the volume of both lungs, in three subgroups of pneumonia.

**Lesion volume Both lungs (%)** was significantly higher in the atypical (median 11.04%) and the COVID-19 (median 12.41%) subgroups compared to the bronchopneumonia (median 5.12%) subgroup (Figure 3). Partial overlap between the three pneumonia subgroups was present.

**Figure 4:**
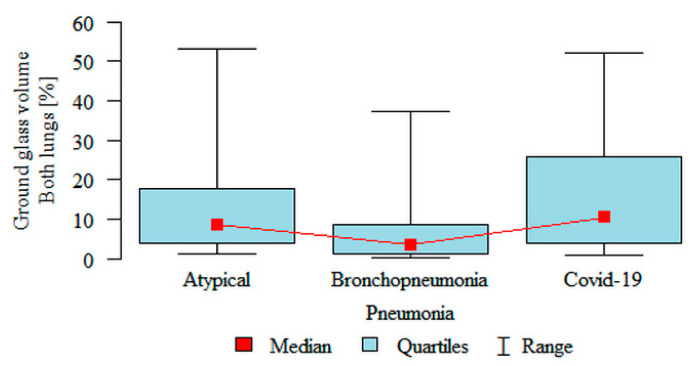
Comparison of the volume of “ground glass” as a percentage of the volume of both lungs, in three subgroups of pneumonia.

**Ground glass volume Both lungs (%)** was significantly higher in the atypical (median 8.61%) and the COVID-19 (median 10.41%) subgroups compared to the bronchopneumonia (median 3.42%) subgroup (Figure 4). Partial overlap between the three pneumonia subgroups was present.

**Figure 5:**
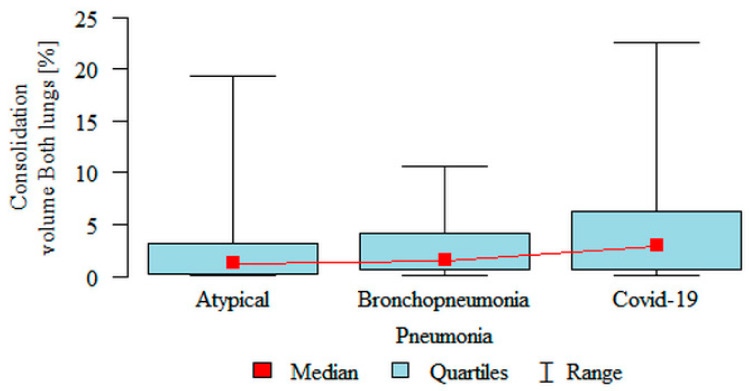
Comparison of the volume of consolidation as a percentage of the volume of both lungs, in three subgroups of pneumonia.

**Consolidation volume Both lungs (%)** was significantly higher in the COVID-19 (median 2.95%) subgroup compared to the atypical (median 1.26%) subgroup (Figure 5). Partial overlap between the three pneumonia subgroups was present.

**Figure 6:**
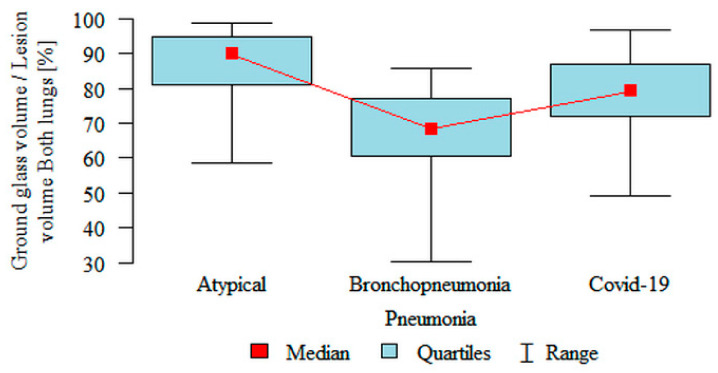
Comparison of the volume of “ground glass” as a percentage of the volume of inflammation (“lesion”) in both lungs, in three subgroups of pneumonia.

**Ground glass volume/Lesion volume Both lungs (%)** was significantly higher in the atypical (median 89.85%) subgroup compared to the COVID-19 (median 79.06%) subgroup, which in turn was significantly higher compared to the bronchopneumonia (median 68.26%) subgroup (Figure 6). However, partial overlap between the three pneumonia subgroups, particularly in COVID-19 pneumonia and atypical pneumonia, was found again.

**Figure 7:**
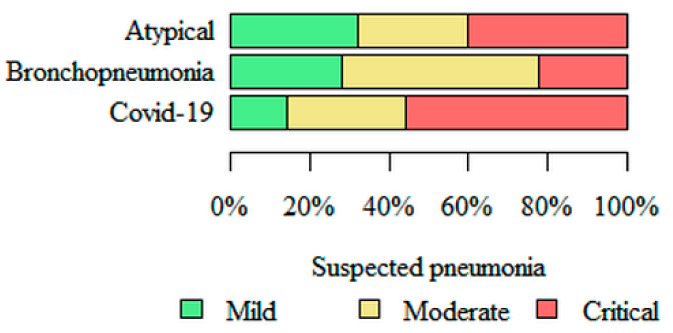
Comparison of the estimated risk of pneumonia, based on HRCT images, in three subgroups of pneumonia.

**Suspected pneumonia**—the greatest, estimated by the software, risk of pneumonia, based on HRCT images, was in the COVID-19 (56% of cases assessed as critical risk) subgroup, and the lowest risk was in the bronchopneumonia (22% of cases assessed as critical risk) subgroup (Figure 7).

The results of the statistical analysis are summarized in Table 1.

## 4. Discussion

In recent years, artificial intelligence technology has found more and more applications in the diagnostics of lung diseases, including lung nodules [3], emphysema [4] and lung cancer [13].

After the outbreak of the COVID-19 pandemic with the dominant lung clinical symptoms, research on the use of artificial intelligence technology in the assessment of chest CT in such patients was quickly launched [14,15,16,17,18].

In our study, the volume of “ground glass” as a percentage of the volume of inflammation in both lungs was significantly higher in the atypical (median 89.85%) subgroup compared to the COVID-19 (median 79.06%) subgroup, which in turn was significantly higher compared to the bronchopneumonia (median 68.26%) subgroup. However, we found partial overlap, particularly between the COVID-19 pneumonia and the atypical pneumonia subgroups.

Li [5] developed a deep learning model—a detection neural network—to extract visual features of COVID-19 from chest CT. Using community acquired pneumonia (CAP) and other non-pneumonia CT exams as a control group, they obtained a sensitivity of 90%, a specificity of 96% and an AUC (area under the receiver operating characteristic curve) of 0.96 for detecting COVID-19. However, they noted the overlap in the chest CT imaging findings of all viral pneumonias and other chest diseases, encouraging a multidisciplinary approach to the final diagnosis. It is similar to our observation of overlap as above.

Duzgun [19] even calls COVID-19 pneumonia “the great radiological mimicker”, emphasizing that CT findings in such pneumonia may be non-specific and variable during the disease course, resembling numerous infectious and non-infectious diseases, including pulmonary edema, hemorrhage, neoplasms, organizing pneumonia, pulmonary alveolar proteinosis, sarcoidosis, pulmonary infarction, interstitial lung diseases and aspiration pneumonia. It also confirms our finding of partial overlap.

Bai [20] assessed the performance of six radiologists in differentiating COVID-19 from viral pneumonia at chest CT. Sensitivity ranged from 72% to 94%, and specificity demonstrated a high variation (24–100%). CT features more commonly in COVID-19 pneumonia than in non-COVID-19 viral pneumonia, including peripheral distribution, ground-glass opacity, fine reticular opacity, vascular thickening and reverse halo sign. In another study, Bai [6] demonstrated that the use of artificial intelligence assistance improved the accuracy in distinguishing COVID-19 pneumonia from pneumonia of other causes on chest CT scans. Without artificial intelligence prediction, six radiologists had an average accuracy of 85%, an average sensitivity of 79% and an average specificity of 88%. Assisted by the artificial intelligence, the radiologists achieved an accuracy of 90%, a sensitivity of 88% and a specificity of 91%. The software used in our study was not trained to literally distinguish COVID-19 pneumonia from pneumonia of other causes, but to automatically detect pulmonary lesions and assess their structure. Therefore, we could not calculate sensitivity and specificity to directly compare our results with the values presented by other authors as above. 

Caruso [21] performed quantitative analysis in discriminating COVID-19 from non-COVID-19 patients with chest CT suggestive for interstitial pneumonia. Lung quantification in liters showed significant differences between COVID-19 and non-COVID-19 patients for “ground glass” opacities (GGO) (0.55 ± 0.26 L vs. 0.43 ± 0.23 L) and fibrotic alterations (0.05 ± 0.03 L vs. 0.04 ± 0.03 L). It is difficult to compare these values with our results because Caruso used absolute volumes, and we used relative “ground glass” volumes—as a percentage of the volume of both lungs or as a percentage of the volume of inflammation. In our study, “ground glass” volume as a percentage of the volume of inflammation in the COVID-19 subgroup was significantly higher compared to the bronchopneumonia subgroup, but, contrary to Caruso’s results, lower compared to the atypical subgroup.

Li [7] presented the spectrum of chest CT manifestations in COVID-19 infection, taking into account the temporal progression of the disease, with the distinction of four phases. In the early phase, the lesions are limited to single or multiple areas of nodular or patchy ground glass lesions. In the progressive phase, the number, extent and density of lesions increase significantly, with coexistence of ground glass areas and consolidations. In the severe phase, peaking around 14 days after the onset of the disease, diffuse infiltration of all segments of the lungs (“white lung”) is visible. Finally, in the dissipative phase, gradual absorption of the lesions is observed, leaving a few cord-like high-density structures, indicative of fibrosis. Thus, ground glass areas, commonly considered typical symptoms of COVID-19 infection, are observed alone only in the early phase—later accompanied by consolidations and diffuse infiltrations. In our study, the patients with pneumonia in the course of COVID-19 infection were examined by chest CT in different phases of the disease. That may explain why the volume of consolidation as a percentage of the volume of both lungs was even the highest in COVID-19 compared to the atypical and the bronchopneumonia subgroups.

In our work, we did not assess the clinical severity of pneumonia, correlated with the volume of parenchymal lesions. It is therefore an important bias that should be taken into account.

Interestingly, quantification of COVID-19 pneumonia lesions on CT images, automatically calculated using artificial intelligence algorithms, can early and non-invasively predict the progression to the severe form [22]. It provides a promising prognostic indicator for further clinical management of COVID-19 patients. In patients with confirmed COVID-19 infection, such analysis may be a valuable method of objective assessment of the dynamics of pulmonary lesions [23]. We plan to address this topic in our future research.

## 5. Conclusions

The final results are:

- The volume percentage of inflammation and the volume percentage of “ground glass” were significantly higher in the atypical and the COVID-19 subgroups compared to the bronchopneumonia subgroup;
- The volume percentage of consolidation was significantly higher in the COVID-19 subgroup compared to the atypical subgroup;
- The percentage of “ground glass” in the volume of inflammation was significantly higher in the atypical subgroup compared to the COVID-19 subgroup, which in turn was significantly higher compared to the bronchopneumonia subgroup.

It confirms the conclusion that HRCT chest images, analyzed automatically by artificial intelligence software, taking into account the structure including “ground glass” and consolidation, significantly differ in three subgroups: COVID-19 pneumonia, bronchopneumonia and atypical pneumonia.

However, the partial overlap found, particularly between the COVID-19 pneumonia and the atypical pneumonia subgroups, may limit the usefulness of automatic analysis in differentiating the etiology.

## Figures and Tables

**Table 1:** The results of statistical analysis of parameters reported by the software after automatic assessment of HRCT images, in three subgroups of pneumonia.

Parameter		Pneumonia			*p*
		Atypical (*N* = 50) - A	Broncho- pneumonia (*N* = 50) - B	COVID-19 (*N* = 50) - C	
Lesion volume Both lungs (%)	Mean ± SD	16.68 ± 17.02	8.96 ± 10.33	21.18 ± 19.66	*p* = 0.001
	Median	11.04	5.12	12.41	
	Quartiles	4.72–22.04	1.88–12.45	5.01–32.55	C, A > B
Ground glass volume Both lungs (%)	Mean ± SD	14.05 ± 14.05	6.22 ± 7.71	15.87 ± 14.55	*p* < 0.001
	Median	8.61	3.42	10.41	
	Quartiles	3.78–17.85	1.35–8.51	3.97–25.88	C, A > B
Consolidation volume Both lungs (%)	Mean ± SD	2.63 ± 3.88	2.75 ± 2.91	5.31±6.35	*p* = 0.05
	Median	1.26	1.54	2.95	
	Quartiles	0.26–3.19	0.62–4.11	0.64–6.34	C > A
Ground glass volume/Lesion volume Both lungs (%)	Mean ± SD	86.28 ± 10.65	67.74 ± 12.4	78.15 ± 12.12	*p* < 0.001
	Median	89.85	68.26	79.06	
	Quartiles	81.22–94.89	60.68–77.26	72.12–86.95	A > C > B
Suspected pneumonia	Mild	16 (32%)	14 (28%)	7 (14%)	*p* = 0.005
	Moderate	14 (28%)	25 (50%)	15 (30%)	
	Critical	20 (40%)	11 (22%)	28 (56%)	

## Data Availability

The data presented in this study are available on request from the corresponding author. The data are not publicly available due to the hospital procedures.
